# Plasma cholinergic markers are associated with post-stroke walking recovery—revisiting the STROKEWALK study

**DOI:** 10.3389/fneur.2025.1568401

**Published:** 2025-05-30

**Authors:** Sumonto Mitra, Taher Darreh-Shori, Erik Lundström, Staffan Eriksson, Tommy Cederholm, Maria Eriksdotter, Birgit Vahlberg

**Affiliations:** ^1^Division of Clinical Geriatrics, Department of Neurobiology, Care Sciences and Society (NVS), Karolinska Institutet, Center for Alzheimer Research, Huddinge, Sweden; ^2^Department of Medical Sciences, Neurology, Uppsala University, Uppsala, Sweden; ^3^Department of Public Health and Caring Sciences, Geriatrics, Uppsala University, Uppsala, Sweden; ^4^Centre for Clinical Research, Sörmland, Uppsala University, Eskilstuna, Sweden; ^5^Department of Community Medicine and Rehabilitation, Physiotherapy, Umeå University, Umeå, Sweden; ^6^Department of Public Health and Caring Sciences, Clinical Nutrition and Metabolism, Uppsala University, Uppsala, Sweden; ^7^Theme Inflammation and Aging, Karolinska University Hospital, Huddinge, Sweden

**Keywords:** brain-derived neurotrophic factor (BDNF), stroke, cholinergic index, exercise, rehabilitation

## Abstract

**Introduction:**

Optimizing post-stroke rehabilitation strategies remains imperative for improving patient outcomes. Physical exercise, including outdoor walking, represents a promising intervention; however, its clinical efficacy, along with the utility of SMS-guided instructions to support adherence, requires further investigation. This study aims to elucidate the association of BDNF levels and cholinergic markers in the plasma of patients with previously reported post-stroke walking recovery (STROKEWALK study).

**Methods:**

Post-stroke patients were randomly selected to receive SMS-guided exercise instructions (intervention group, *n* = 31) or not (control group, *n* = 31) at the time of stroke (baseline) and continued for the next 3 months. Plasma samples were collected at baseline (*n* = 28) and at 3-month follow-up (*n* = 28) and analyzed for Brain-Derived-Neurotrophic-Factor (BDNF) protein as a primary outcome. Secondary outcomes included enzyme activities of choline acetyltransferase (ChAT) and Butyrylcholinesterase (BChE), and the six-minute walking test (6MWT), which was assessed at the same time as the plasma sampling.

**Results:**

A significant decline in BDNF was observed at 3 months in the total population (*n* = 56), primarily driven by the control group. Stratifying groups as intervention or control displayed no significant difference in BDNF protein levels, nor in ChAT or BChE activities at baseline or at 3-month follow-up, except for a significant correlation between BChE and Body-Mass Index (BMI). Patient stratification based on 6MWT performance displayed higher BDNF levels in the intervention group versus the control group, especially among females but not males. Females showed higher BChE than the males in the control group, but not in the intervention. Interestingly, the change in ChAT activity and cholinergic index (ChAT/BChE) from baseline to follow-up is significantly correlated with 6MWT performance.

**Discussion:**

We conclude that SMS-guided exercise training improves post-stroke walking performance (6MWT) which attenuates the decline in BDNF levels. Cholinergic function correlates with improved walking performance and could be a useful marker to evaluate rehabilitation outcomes.

## 1 Introduction

Stroke is a serious life-threatening medical condition and is the second most common cause of mortality and disability worldwide (1). Among various risk factors involved in later-life stroke incidence, several modifiable lifestyle factors have been reported recently (2). A sedentary lifestyle has been linked to both stroke incidence and outcome (3, 4), thus therapeutic programs including physical activity intervention are currently employed (5–7). Recently, great interest has been reported in utilizing web-based or mobile-based applications to support stroke patient’s rehabilitation (8). Although physical exercise and activity may have meaningful benefits for stroke patients (9), more knowledge on underlying pathways is needed to evaluate their clinical benefits (10–12).

We have previously performed the STROKEWALK Study (13) where patients received text-message-guided physical exercise instructions for post-stroke training (treatment group) or not (control group). Between-group analysis revealed that 3 months of daily text messages (SMS) containing training instructions significantly improved walking performance (as measured by the six-minute walk test, 6MWT) and lower body strength in favor of the SMS treatment group (13, 14). The present study uses secondary analysis from the STROKEWALK study and aims to explore the molecular changes in plasma samples in the intervention and control groups.

Since physical exercise has been associated with increased plasma levels of brain-derived neurotrophic factor (BDNF) (15), which has neurotrophic effects in the brain (16), BDNF protein levels were estimated. Similarly, exercise is also associated with increased cholinergic input in the hippocampus and cortical regions of the brain (17), wherein cholinergic pathways modulate cognitive pathways (18). In addition, cholinergic pathways have important roles such as the anti-inflammatory and functional recovery of neurons (19, 20), and may aid in post-stroke recovery in the brain (21) and play crucial role in modulation neuromuscular junctions (22) in the periphery, which could be critical for post-stroke physical activity. Therefore, the plasma cholinergic markers choline acetyltransferase (ChAT) and butyrylcholinesterase (BChE) were analyzed. In this exploratory study, we aimed to study whether effects from the text messaging-guided training could induce changes in BDNF and cholinergic markers. We hypothesized that physical exercise would enhance walking performance and increase BDNF levels and that improvements in 6MWT outcomes would correlate with elevated cholinergic function in patient plasma.

## 2 Methods

### 2.1 Design and participants

Design, recruitment, determination of sample size, and intervention have been previously described (13). Recruitment started on November 1st, 2016; the last follow-up assessment was performed on December 19th, 2018. Briefly, this study included participants who were aged 18 or older, had a verified stroke (CT scan, infarction, or intracerebral hemorrhage with first or recurrent event), and were planning to be discharged home to independent living with access to a mobile phone. They also had no cognitive impairment (MoCA ≥ 23 points), a Modified Rankin scale of 2 or less, and could perform a six-minute walk test (6MWT) at discharge, with or without walking aids (13). Exclusion criteria included subarachnoid hemorrhage, uncontrolled hypertension, untreated arrhythmias, unstable cardiovascular conditions, dementia, severe aphasia, psychiatric issues, and difficulty understanding instructions. Randomization and allocation concealment were previously reported (13). Patients with a transient ischemic attack (TIA) were excluded from the present study.

Patients were classified into two subgroups for data analysis: (1) control or intervention group, based on whether they received SMS-guided training instructions; and (2) based on improvement in the 6MWT, categorized less than 34 m or 34 m or more, since a clinically significant improvement in physical recovery was observed in post-stroke patients who exceeded this threshold (13). Gender differences were also analyzed at multiple points to assess the potential role of sex in post-stroke recovery following our intervention (23). Individuals with stroke were included in the study a median of 5 days (IQR = 5) after hospital admission. Of these patients, 87.5% (*n* = 49) had a cerebral infarction (CI) and 12.5% (*n* = 7) had an intracerebral bleeding (ICH) [SMS group: CI: 89.3% (*n* = 25) and ICH: 10.7% (*n* = 3)].

Non-fasting blood samples were collected in heparinized vials at the hospital at the time of the stroke and during follow-up after 3 months. Plasma samples were collected at the same time as the 6MWT (meters) was assessed. The samples were frozen at −80°C until analysis.

This study is a secondary analysis of participants from a randomized controlled trial performed at the Uppsala University Hospital in Sweden. The original study was performed according to the Helsinki Declaration, and ethical approval was obtained from the regional Ethical Review Board of Uppsala University Hospital, Sweden: Dnr: 2015/550 and Dnr 2020/01087 for analyses of BDNF and cholinergic biomarkers. The study was registered with ClinicalTrials.gov (NCT 02902367).

### 2.2 Primary and secondary outcomes

The primary outcome variable in the present study was the concentration of brain-derived neurotrophic factor (BDNF), measured in the blood (plasma) at baseline and 3-month follow-up. Secondary outcomes were changes from the baseline and 3 months in butyrylcholinesterase (BChE) activity, Choline acetyltransferase (ChAT) activity, and the ChAT/BChE ratio (cholinergic index), measured in blood (plasma).

### 2.3 The intervention

The SMS-intervention group received daily cost-free text SMS^1^ with simple instructions on what and how to exercise as an add-on to standard care for 3 months and comprised three different strategies: (1) 3 months of daily SMS-text messages, (2) training diaries, (3) pedometers for step counts during the first and last week of intervention (13). The text messaging gave instructions on how to exercise to increase outdoor walking performance and lower body strength, without the possibility of texting back for help or advice. The outdoor walking gradually increased in walking time and the perceived/subjective intensity level increased from moderate to strenuous intensity by the third month. Moreover, the number of repetitions of the sit-to-stand exercise also gradually increased.

Control patients received standard care without any restrictions on physical activity, including exercise.

### 2.4 Randomization

The allocation to either intervention or control group was based on randomization, a 1:1 ratio, stratified by gender as previously reported (13).

### 2.5 Outcome assessments

#### 2.5.1 Walking performance—the six-minute walking test

The maximal walking distance during 6 min over a 30-meter course was measured by the six-minute walking test (6MWT) and changes from baseline to three-month follow-ups were registered (24). The participants were instructed to walk at their maximum possible speed.

#### 2.5.2 Estimation of plasma BDNF levels

Total BDNF levels were measured in plasma using the DuoSet BDNF ELISA kit (DY248, R&D Systems) following the manufacturer’s protocol, with minor modifications wherein the detection system was replaced with alkaline phosphatase (prepared in reagent diluent, 1:10000; #11093266910, Roche Diagnostics) which was added for 2 h at RT. Samples were diluted 10 times in reagent diluent (1% BSA in PBS, 0.01% sodium azide, 0.22 μm filtered, pH 7.4) and measured twice in triplicates, to calculate the mean value. Absorbance data was kinetically read at 405 nm, every 5 min for a total duration of 1 h, using a spectrophotometer (Infinite M1000, Tecan).

#### 2.5.3 Analysis of ChAT activity

Analysis of total ChAT activity was performed in 300 times diluted plasma samples as described previously (25). Briefly, plasma samples were prepared in dilution buffer (10 mM TBS, 0.05% Triton X-100, 1 mM EDTA, pH 7.4) and immediately processed. Each sample was applied in 384-well plates (#464718, Nunc MaxiSorp) in two formats in triplicates—native (10 μL/well of the actual sample) or denatured. The denatured sample was obtained by heating one aliquot of the actual diluted sample in a thermal cycler (3 × 8 min at 98°C), chilled briefly on ice, and centrifuged at 2,000 rpm for 2 min, and 10 μL supernatant was plated per well in triplicate.

Reaction was initiated on the native and denatured samples by adding 40 μL/well of cocktail A which contained 62.5 μM coenzyme-A lithium salt, 1.25 U/mL phosphotransacetylase, 14.78 mM lithium potassium acetyl-phosphate, 37.5 μM choline chloride and 0.075 mM eserine hemisulfate, prepared in dilution buffer. The plate was sealed and incubated at 38°C for 1 h. To calculate ChAT activity, a standard curve was prepared by serially diluting choline chloride starting from 100 μM to 0.78 μM prepared in dilution buffer and processed in triplicates (50 μL/well) in the same plate.

Subsequently, all the wells received 25 μL of cocktail B which was prepared in 50 mM PBS containing 0.93 U/mL choline oxidase, 1 U/5,000 μL Streptavidin-HRP, 3 mM 4-aminoantipyrine, 6.3 mM phenol, pH-7.6. Absorbance was immediately read at 500 nm for 1 h. ChAT activity was calculated by assessing the difference in choline concentration between denatured versus native samples as calculated using the choline chloride standard curve (pMole/min/mL samples).

#### 2.5.4 Analysis of BChE activity

Analysis of the total BChE activity in the plasma samples was performed as previously described (26). Briefly, plasma samples (intervention and control groups) were diluted 400 times with dilution buffer containing 10 mM tris, 0.1% BSA, 1 mM EDTA, and 0.05% Triton X-100, pH 7.4. BChE activity was measured using 50 μL/well of samples which was then mixed with 25 μL/well of 3X master mix containing 15 mM butyrylthiocholine iodide, 1.2 mM 5,5′-dithiobis (2-nitrobenzoic acid) (DTNB), 1 μM of specific AChE inhibitor BW280C51, prepared in 50 mM sodium-potassium phosphate buffer, pH 7.4. The plates were immediately read in a spectrophotometer (Infinite M1000, Tecan) kinetically at 412 nm absorbance with 1 min intervals for a total duration of 20 min.

#### 2.5.5 Calculation of cholinergic index

The cholinergic index was calculated as a ratio of the total ChAT activity (nMole/min/mL) to the total BChE activity (nMole/min/mL). Cholinergic index indicates the equilibrium of acetylcholine (ACh) availability which may influence various target pathways (22, 27).

#### 2.5.6 Statistical analysis

No *a priori* power analysis was performed in this study and statistical analyses are based on complete case analysis. The baseline clinical characteristics were compared using Pearson’s chi-squared test (*χ*^2^). The plasma biomarker data were transformed using natural logarithms (Ln). The within-group changes over time were analyzed by the Wilcoxon signed-rank test. The differences between groups at baseline or the three-month follow-up and changes from baseline to follow-up were tested by the nonparametric Mann–Whitney U test. The use of non-parametric vs. parametric is a precautionary measure that we adopted to avoid any possible effect of outliers given the exploratory nature of the study and the relatively small number of subjects in the subgroups. All data are then illustrated as relative percentage change at follow-up compared to baseline using Notched Box Plots, where the notches represent a 95% confidence interval (CI) around the median. All other characteristics are the same as the conventional Box Plots.

The correlation analyses were performed using Spearman Rank correlation analyses and were visualized using simple regression analyses on Ln transformed data. In some cases, when deemed appropriate, we logarithmically (Ln) transformed the data to ensure their normal distribution. In these cases, we found that whether the Ln-transformed data were tested using parametric or non-parametric tests, there were no differences in the statistical tests results. Statistical significance was examined using a two-tailed test with a *p* value set at *p* < 0.05.

## 3 Results

### 3.1 Patient demographics and study outcomes

**Figure 1:**
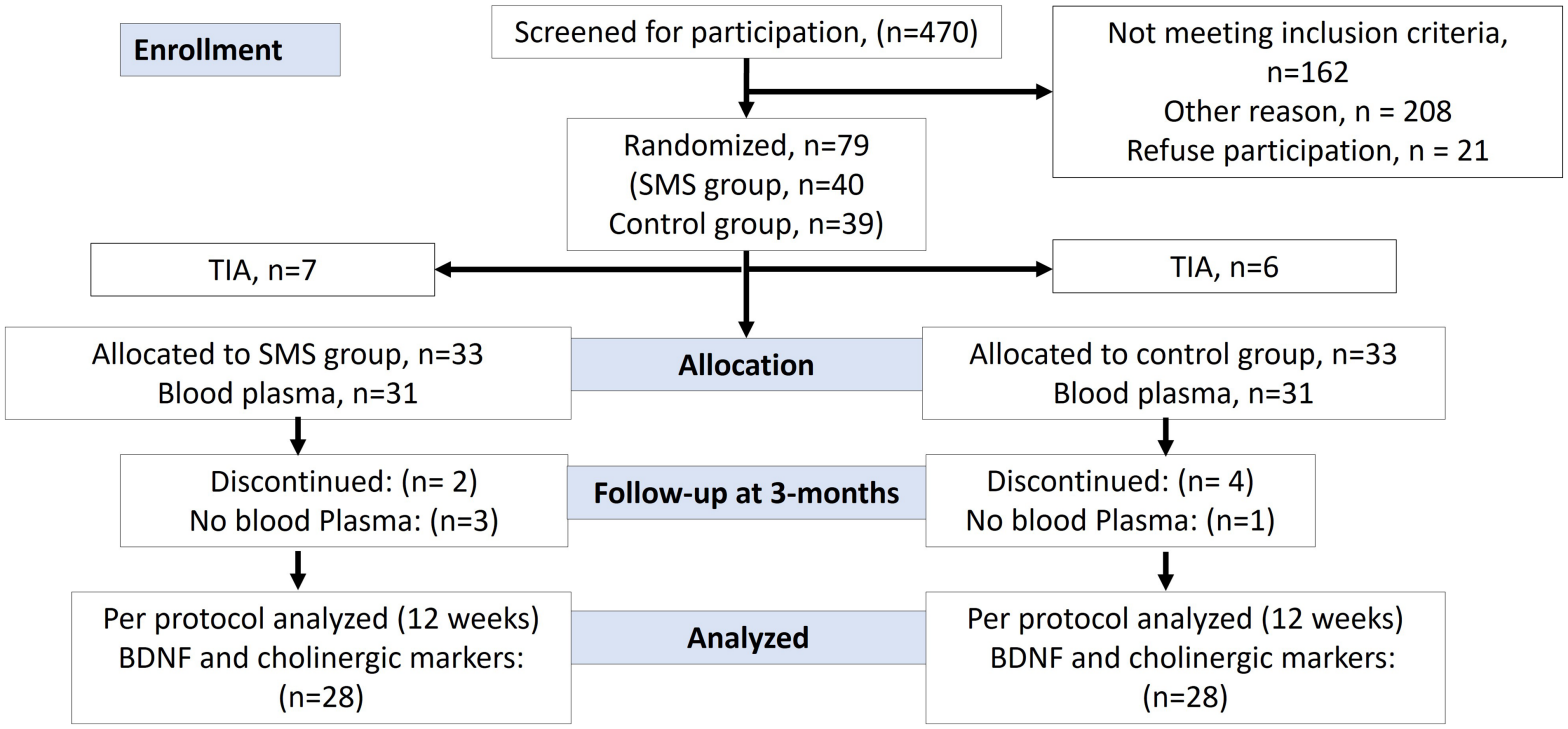
Flow chart of the study participants through the phases of the randomized controlled trial. Individuals were randomized within 1 week after baseline assessment. Data were collected at Uppsala University Hospital, Sweden between November 1st, 2016, and December 18th, 2018. Analysis of BDNF protein levels and cholinergic enzyme activity from plasma.

The inclusion and retention process of patients is shown as CONSORT mapping in Figure 1. Of 79 individuals participating in the original study, 62 individuals with successful baseline plasma collection were randomized equally to the SMS intervention and the control group (13 TIA patients were excluded from the present analysis). Subsequently, in total 56 participants completed the study with successful plasma collection at 3-month follow-up, which was analyzed in this study (*n* = 28 in each group). Due to logistic reasons, some individuals were not able to provide the study with blood samples. The clinical characteristics at baseline are provided in Table 1. In total, the mean (SD) age was 64 (9.4) years, and 19 (33.9%) participants were women. Forty-nine individuals suffered an ischemic stroke (87.5%), and seven (12.5%) individuals suffered an intracerebral hemorrhage. According to the training diaries, adherence to the recommended SMS interventions ranged from 59 to 100% (13). The mean increase in steps per day was 2,084 (SD = 2,538). Among the 26 participants receiving the SMS, 6 experienced a decrease in their daily step count.

**Table 1:** Baseline clinical characteristics of the study population with minor stroke allocated to the text messaging or control group.

Variables	Missing value	Text messaging (*n* = 28)	Missing value	Control group (*n* = 28)
Gender, female, *n* (%)	0	11 (39.3)	0	8 (28.6)
Age (years), mean (SD)	0	63.3 (9.8)	0	64.6 (8.8)
Body mass index, kg/m^2^, mean (SD)	0	27.5 (4.5)	0	26.7 (3.6)
Body mass index, kg/m^2^, classes, *n* (%)				
Normal weight: <25	0	8 (28.6)	0	9 (32.1)
Overweight: 25–29.9	0	13 (46.4)	0	15 (53.6)
Obesity, class I: 30–35	0	5 (17.9)	0	3 (10.7)
Obesity class II: 35.1–39.9	0	2 (7.1)	0	1 (3.6)
Obesity, class III: ≥40	0		0	
Montreal cognitive assessment scale, Md (IQR)	3	27 (4)	5	26 (4)
Saltin-Grimby physical activity level, *n* (%)				
Sedentary	0	2 (7.1)	0	6 (21.4)
Light physical activity	0	18 (64.3)	0	17 (60.7)
Moderate/high physical activity	0	8 (28.6)	0	5 (17.9)
Modified Rankin scale, *n* (%)				
0	0	3 (10.7)	0	-
1	0	18 (64.3)	0	22 (78.6)
2	0	7 (25.0)	0	6 (21.4)
Six-minute walk test (6MWT), meters, mean (SD)	0	475 (99)	0	470 (125)
Cerebral infarction, *n* (%)	0	25 (89.3)	0	24 (85.7)
Intracerebral hemorrhage, *n* (%)	0	3 (10.7)	0	4 (14.3)
Thrombolysis, *n* (%)	0	2 (7.1)	0	1 (3.6)

**Figure 2:**
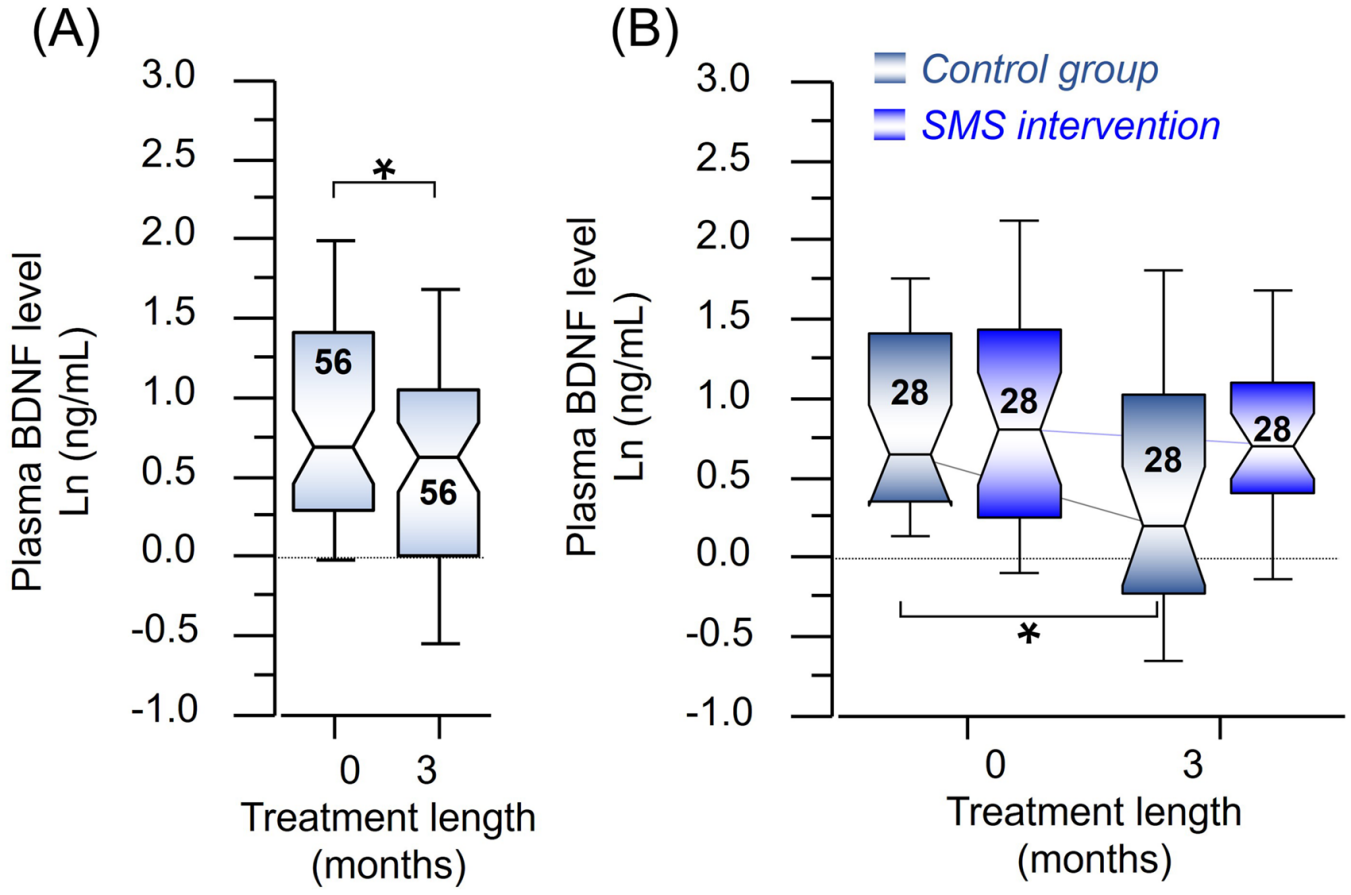
**(A)** Comparison of the level of BDNF between baseline and the follow-up in the whole study population (Ln, *n* = 56). **(B)** Time-dependent changes in the plasma BDNF levels when groups were stratified as control or intervention subgroups. The digits in the boxes represent the number of subjects in each subgroup. The notches of the boxes represent a 95% confidence interval around the median value. * All *p* values were <0.02.

A significant post-stroke reduction in BDNF levels was observed (*p* < 0.02, 25% reduction) when all samples (*n* = 56, Ln data) were compared between baseline versus 3-month follow-up (Figure 2A). A sub-group analysis revealed a significant reduction in the control group (Ln, *p* < 0.02, 39% reduction), but not in the intervention group (Ln, *p* < 0.40, 12% reduction) (Figure 2B), signifying a positive role of physical exercise in resisting post-stroke decline in BDNF levels. There were no differences between the groups when analyzed on actual data (mean ± SD) (Table 2), except significant reduction in BDNF levels in the control group at 3-month follow-up as also shown in Figure 2B. There was no association between changes in BDNF and changes in the 6MWT at 3 months, *r* = −0.16, *p* = 0.25 (or any of the cholinergic markers) considering the intervention or control groups, respectively (Table 2).

**Table 2:** Within-group and between-group differences of BDNF and plasma cholinergic markers in patients with minor stroke at the start of the intervention and after 3 months.

Stroke	Intervention group (*n* = 28)	*n* = 28	*p*-value	Control group (*n* = 28)	*n* = 28	*p*-value	Median estimate (95%CI)	*p*-value
	Baseline	3-month		Baseline	3-month		Between-group differences	
BDNF, ng/mL, mean (SD)	3.4 (3.0)	2.6 (1.7)	0.36	3.0 (2.6)	2.4 (2.4)	0.013	−0.3 (−1.3 to 1.1)	0.54
ChAT activity, nmol/min/mL, mean (SD)	278.5 (209.4)	253.5 (239.3)	0.43	247.2 (128.3)	245.0 (108.2)	0.83	29.8 (−56.3 to 120.5)	0.50
BChE, nmol/min/mL, mean (SD)	2384.7 (577.1)	2397.7 (576.6)	0.84	2208.7 (554.2)	2214.5 (493.0)	0.78	−29.3 (−266.8 to 186.3)	0.79
ChAT/BChE ratio, mean (SD)	0.119 (0.071)	0.108 (0.084)	0.54	0.118 (0.060)	0.116 (0.058)	0.94	0.012 (−0.03 to 0.06)	0.52

BDNF, brain-derived neurotrophic factor; BChE, butyrylcholinesterase; ChAT, Choline acetyltransferase; SD, Standard deviation.

The table displays actual data as mean ± SD. The within-group differences were analyzed with Wilcoxon’s Signed Ranks Tests. Differences between groups (changes from baseline to follow-up) were analyzed by the Mann–Whitney U test. The median estimate was calculated using the Hodges-Lehmann test. CI indicates confidence interval.

At baseline, BChE concentration correlated with Body Mass Index (BMI), *r* = 0.49, *p* < 0.0003 (Supplementary Figure 1A). A similar correlation was observed at 3 months with *r* = 0.54 and *p* < 0.0001, including both the control and intervention groups, respectively (Supplementary Figure 1B).

### 3.2 Sub-group analysis showed increased plasma BDNF levels in the intervention group

**Figure 3:**
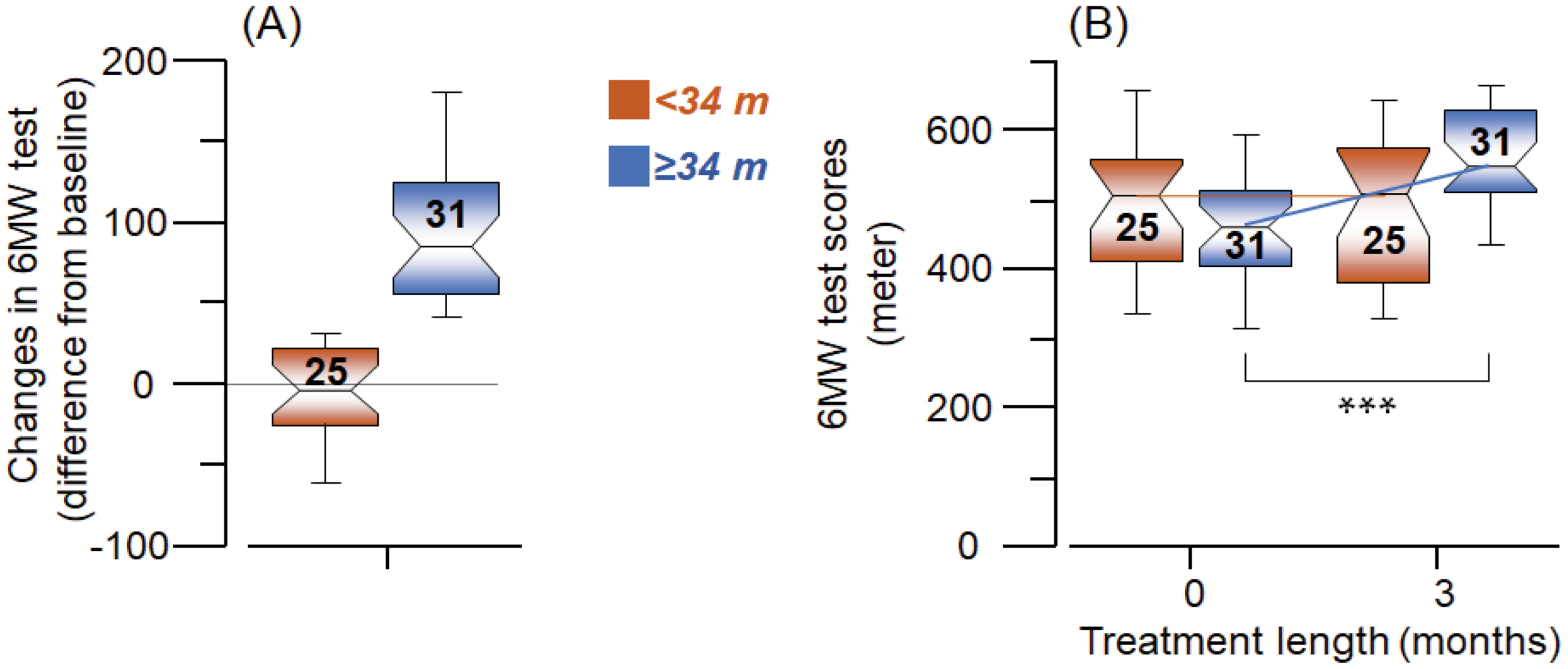
**(A)** Grouping the patients based on walking performance after 3 months of treatment assessed by scoring their ability to walk less than 34 meters (<34 m) and more than or equivalent to 34 meters (≥34 m), wherein the figure shows the extent of change from the baseline assessment to follow-up at 3 months. **(B)** The six-minute walking test (6MWT, meters) groups as defined in **(A)**, showing equivalent walking performance at baseline (red vs. blue) but a group of patients went on to improve their walking performance at follow-up (blue). The digits in the boxes represent the number of subjects. The notches of the boxes represent a 95% confidence interval around the median value. *** *p* < 0.02.

We wanted to explore whether improvement in clinically meaningful walking capacity measured by 6MWT could be related to changes in the plasma biomarkers. We sub-grouped the patients based on changes in walking performance, i.e., 6MWT scores, in terms of their ability to walk over (≥34 m, *Improved group*) or below 34 m (<34 m, *Unchanged group*) compared to their baseline performance (Figure 3), based on previous observation (28).

**Figure 4:**
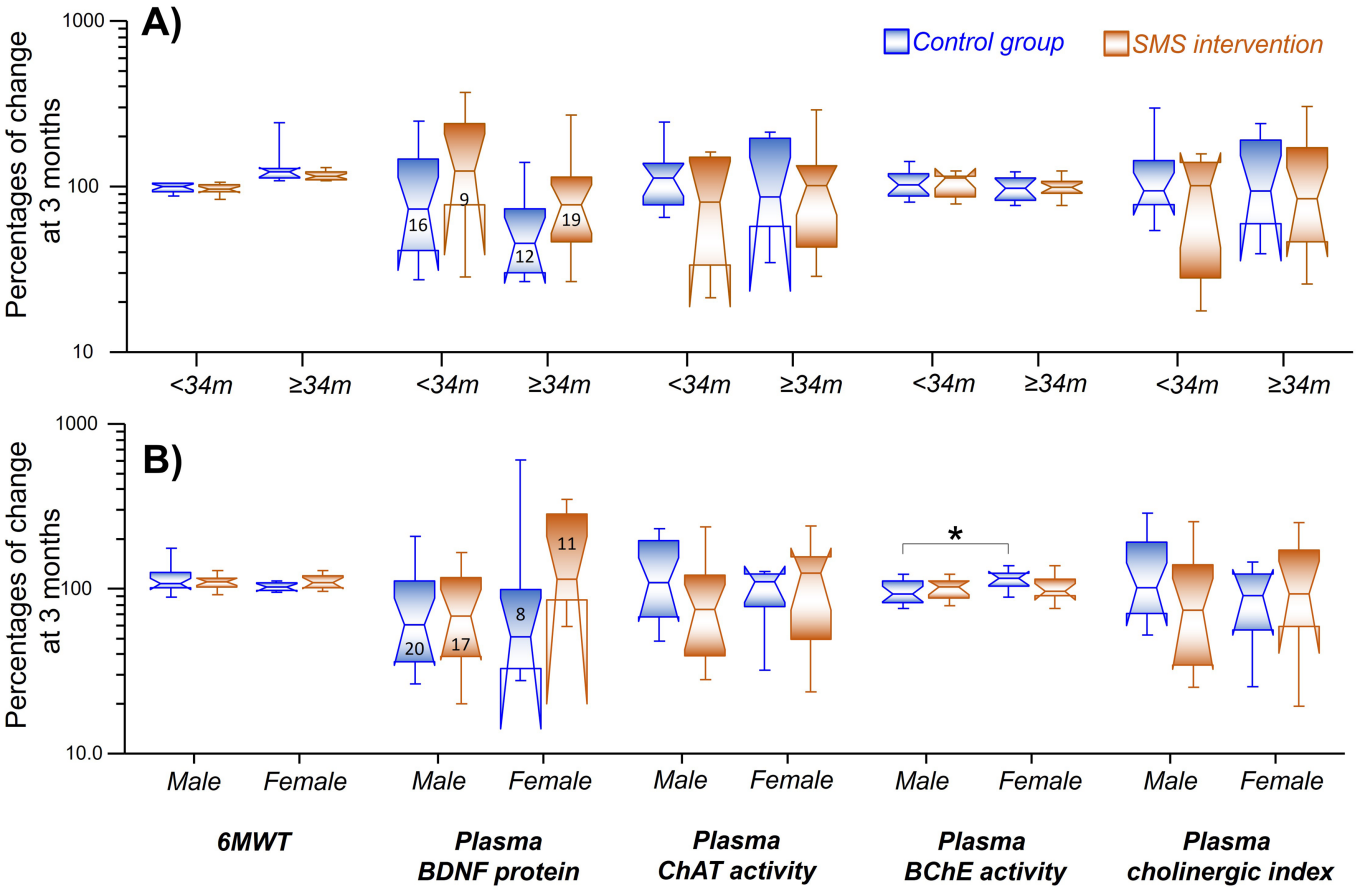
**(A)** Changes relative to baseline levels of the assessed biomarkers in relation to the treatment outcomes subgroups. Grouping was done as control (blue) or intervention (red), with subsequent stratification based on whether the outcomes on 6MWT (X-axis). **(B)** Changes in relative to baseline levels of the assessed biomarkers (X-axis), wherein the groups were stratified on the basis of gender (between men and women) among the treatment groups. *p < 0.05. The digits in the boxes represent the number of subjects applicable to all parameters. The notches of the boxes represent 95% confidence interval around the median value. The color coding applies to both the figures.

Using this subgrouping, the SMS intervention group tended to have a higher plasma level of BDNF compared to the controls (Figure 4A, statistically not significant, *p* < 0.07) when it was analyzed among the *Improved group* (≥34 m). The corresponding changes among the *Unchanged group* (<34 m) were just numerically higher in the SMS intervention group (~23%, Figure 4A, *p* < 0.34).

Further analyses did not show any differences in relative levels of ChAT activity, BChE activity, or the estimated cholinergic index in plasma between the main groups or the treatment outcome subgroups (Figure 4A).

### 3.3 Gender differences in BDNF and cholinergic markers

The plasma level of BDNF tended to be higher among women in the SMS-intervention group (~18%) compared to women in the control group (Figure 4B, statistically not significant, *p* < 0.07).

There was also a 16% significant difference between the changes in the plasma BChE activity in men versus women in the control group (Figure 4B, *p* < 0.04), but no sex difference was observed in the SMS intervention group.

Further analyses did not show any differences between men and women with regard to the changes in the 6MWT or the relative levels of the plasma ChAT activity or the estimated plasma cholinergic index (Figure 4B).

### 3.4 Plasma ChAT activity predicts and is correlated with post-stroke walking recovery

Analyses of the baseline data revealed no correlations between the actual 6MWT scores (Ln 6MWT) and levels of BDNF or BChE in the plasma baseline samples among the overall study population or the treatment arms of the study (i.e., the control vs. SMS intervention).

However, there was a positive correlation between the ChAT activity in plasma and 6MWT at baseline (rho *=* 0.35, *p* < 0.05, *n* = 32), among *the Improved group* who showed ≥34 m walking improvement as was defined in Figure 3. The correlation was reversed among *the Unchanged group* (i.e., patients who showed <34 m walking improvement) (rho = −0.39, *p* < 0.05, *n* = 28). A similar positive correlation was found regarding the plasma cholinergic index within *the Improved group* (rho = 0.36, *p* < 0.05, *n* = 32), but not among *the Unchanged group* (rho = −0.16, *p* < 0.4, *n* = 28).

Further analyses based on the SMS-intervention and the control subgroups among the *Unchanged group* revealed that the negative correlation between the plasma ChAT activity and the 6MWT results were significant among the SMS intervention group (rho = −0.70, *p* < 0.04, *n* = 10) but not Control (rho = −0.19, *p* < 0.44, *n* = 18). This relationship was reversed among *the Improved group,* i.e., the correlation was not significant among the SMS intervention group (rho = 0.16, *p* < 0.5, *n* = 19) but instead was significant in *the Control* (rho = 0.73, *p* < 0.02, *n* = 12). Similar correlation analyses between the 6MWT results and the plasma cholinergic index showed the same relative relationships.

**Figure 5:**
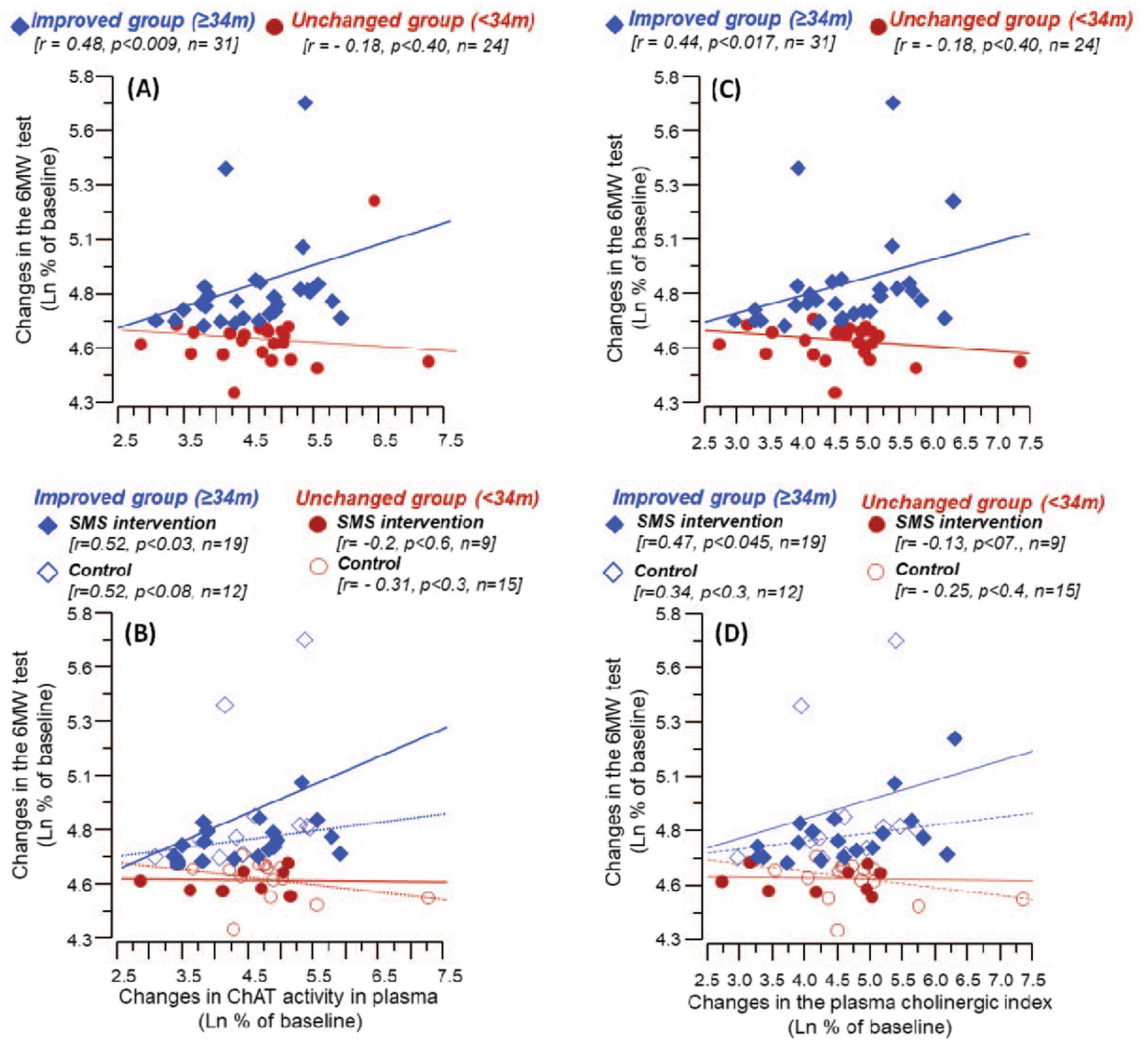
Correlation between changes from baseline levels till 3 months follow-up in 6MWT and the relative levels of ChAT activity and cholinergic index in plasma. **(A)** Shows the correlation between %changes in 6MWT and the plasma ChAT activity among *the Improved and the Unchanged groups (based on improvement on 6MWT at follow-up).* **(B)** Illustrates the corresponding correlations when the *Improved and the Unchanged groups* were further stratified based on the intervention (control or SMS-intervention). Similarly, **(C)** shows the correlation between %changes in 6MWT and the cholinergic index among *the Improved* and *the Unchanged groups*, whereas **(D)** illustrates the corresponding correlations among the intervention and control groups. In **(B)** and **(D)**, solid lines represent correlation among *the intervention group*, while the dashed lines among *the control group*.

The correlation analyses between the absolute changes in 6MWT and the assessed plasma biomarkers at the completion of the study, i.e., the 3-month assessment did not show any significant findings. Nonetheless, analyses on the relative changes from baseline to the 3-month follow-up revealed a significant positive correlation among the *Improved group* between changes in the 6MWT distance and the changes in the activity of ChAT in plasma (Figure 5A). The analyses on the subgroups showed that the correlation was strongest among the SMS intervention group (Figure 5B).

### 3.5 Plasma cholinergic index is correlated with improved post-stroke walking performance

Like ChAT, changes in the cholinergic index showed a significant positive correlation with the changes in the 6MWT result among the Improved group (rho = 0.44, *p* < 0.017, *n* = 31) (Figure 5C). Further subgroup analysis showed that the correlation was strongest among the SMS intervention group (Figure 5D).

There was no significant correlation between changes in the 6MWT result and the changes in the BDNF levels or BChE activity in plasma. The subgroup analyses did not show any significant correlation among the subgroups.

## 4 Discussion

This is one of the first studies to measure cholinergic index among post-stroke patients and we report an association of ChAT activity and cholinergic index with improved walking performance. Interestingly, the decline in BDNF levels post-stroke was significant in the control group but attenuated by the intervention, indicating a beneficial effect of exercise. A gender-specific change in total BDNF level was observed in females who showed increased BDNF in the SMS intervention group as compared to the control group, although they both showed improvement in 6MWT, respectively. However, due to the limited sample size, our findings represent exploratory findings.

Discrepancies remain about changes in BDNF levels at post-stroke follow-up in plasma/serum (29). We report that BDNF levels were significantly reduced post-stroke (Figure 2) until 3 months compared to the baseline but remain within the quantitative range as reported previously (30). BDNF was associated with various post-stroke outcomes, wherein some studies associate BDNF with a specific outcome while most did not find such an association (29, 31). This could be due to the different treatments provided to the patients in various studies included in a meta-analysis, and several technical aspects that might affect serum and plasma BDNF stability prior or during the estimation process (32).

Previous reports utilizing acute and long-term exercise have been reported to increase BDNF levels in the plasma and serum (15), which has a neurotrophic effect in the brain hippocampus (16). Although we did not observe any significant difference between the intervention and the control groups either at baseline or follow-up interval, the decrease in BDNF levels was significant in the control group but not in the intervention group, when compared to baseline levels (Table 2). Although an overall decrease in BDNF levels was observed post-stroke (Figure 2), stratifying post-stroke patients based on 6MWT performance displayed significantly higher BDNF levels in patients who improved most (i.e., ≥34 m) and received the intervention (Figure 4), probably signifying a beneficial role of exercise in reducing the decline in BDNF levels. Higher BDNF was more prominent in females than males when the intervention group was compared to control groups (Figure 4). Whether these improved plasma BDNF levels elevated brain function or affected other bodily functions (33), warrants further research.

Notably, recent studies have highlighted various physiological role of BDNF in the central and peripheral system [reviewed by Miranda et al. (34) and Ichimura-Shimizu et al. (35)]. Among others, cardiovascular risk factors are known to be crucial for exercise-induced secondary prevention strategies in stroke patients [reviewed by D’Isabella et al. (36)], wherein constitutive BDNF-mediated signaling was previously reported to be crucial for normal heart function (37). BDNF is critical in mediating the beneficial effects of exercise induced stress response and regulates myocardial cellular energetics (38) and contributes to reducing post-ischemic heart failure (39). Nonetheless, our data shows that BDNF levels are reduced post-stroke and physical activity could be a good tool to resist the reduction of plasma BDNF levels which may counter post-stroke depression (40) along with cardioprotective effects.

Cholinergic pathways have been reported to be involved in post-stroke recovery in the brain (21) and improve memory function (41). This might be mediated via parasympathetic pathways (42), for which acetylcholine-mediated transmission is an important pre-requisite. Even though ChAT activity went down from baseline to 3-month follow-up in both groups, we found that change in ChAT activity from baseline to follow-up was significantly correlated with the improvement in 6MWT in the *Improved group*, indicating a role of cholinergic pathways in modulating the clinical outcomes (Figure 5). One of the plausible explanations could be the involvement of ACh in regulating inflammation, blood vessel constriction, muscle activity, etc. among many others (43, 44). Patients in our study were followed up to 1-year post-stroke, wherein none but one control patient died during this period.

We also measured the ACh degradative enzyme BChE in plasma, but did not find any difference in BChE activity between the intervention versus control groups at 3-month follow-up, nor when the samples were stratified based on 6MWT performance (Table 2, Figure 4A). We found a gender-specific differential activity, where females in the control group showed significantly increased BChE activity compared to males in the respective group. BChE activity was found to be increased at 3-month follow-up in the intervention group, whereas it decreased in the control group, without reaching any statistical significance when compared to baseline (Table 2). Increased post-stroke BChE has been previously reported to be associated with delirium, mortality prediction, and dementia (45–47). Although BChE activity was found higher in the intervention group compared to the control group (Table 2), SMS intervention reduced BChE activity among females by the end of 3 months (Figure 4B).

Accumulating evidence indicates that ACh, besides its canonical function as a neurotransmitter, plays a key role in regulating various central and peripheral processes, including inflammatory responses and function of various organs (48). Given that stroke inevitably causes acute inflammatory responses which can affect various organs, the aforenoted positive correlation between changes in the clinical outcome and the ChAT activity suggests that the patients who were able to increase ChAT activity or their cholinergic index were successful in favorably modulating the outcome of the acute inflammatory responses, and thereby those patients were better able to clinically recuperate. Please note that in this study, the above mechanism may seem quite speculative. However, it should be noted that in stroke most often the BBB integrity becomes affected, allowing circulating immune cells to readily access the affected stroke sites, and ACh has been known to induce anti-inflammatory effect on immune cells (49).

Our mechanistic hypothesis concerns with an inherent capability of the patients in elevating ACh equilibrium state, most likely depending on the net outcome of a combination of feedback responses, one to acute inflammation as consequence of the stroke and the other to the treatment (physical activity) and/or the patient’s physical condition. For example, we found a positive correlation between the patients’ BMI and the plasma BChE activity both at the baseline and the follow-up (Supplementary material). This might negatively affect the cholinergic index in subjects with high BMI since BChE activity is in the denominator of the ratio of ChAT/BChE (i.e., because BChE degrades ACh). This in turn could reduce the efficacy of such patient’s cholinergic anti-inflammatory response and thereby hamper effectively coping with consequences of an excessive inflammatory response. In addition, in patients with poorer physical condition the balance between the sympathetic and parasympathetic may become unfavorable with regard to a cholinergic anti-inflammatory response due to a reduced vagal input (required to raise the heartrate by sympathetic input). Nonetheless, this is a hypothetical explanation, which is quite speculative.

The calculation of the changes in the plasma cholinergic index involves determining percentages of two ratios between ChAT to BChE activities, at baseline and at the follow-up. This could result in some distortion of the data. However, this is unlikely to be a major issue in the current study given the close agreement between the data presented in Figure 5A vs. Figure 5C or between Figure 5B vs. Figure 5D. In addition, the cholinergic index calculations have been extensively assessed previously (25, 50–55). Stronger correlations of the cholinergic index with 6MWT were observed which is perhaps driven by ChAT activity, which was also found to correlate with 6MWT, respectively (Figure 5). This might be mediated through the anti-inflammatory function of ACh on other aspects of physiology including cardiovascular and vascular pathways, which need to be studied further (43, 44).

## 5 Conclusion

**Figure 6:**
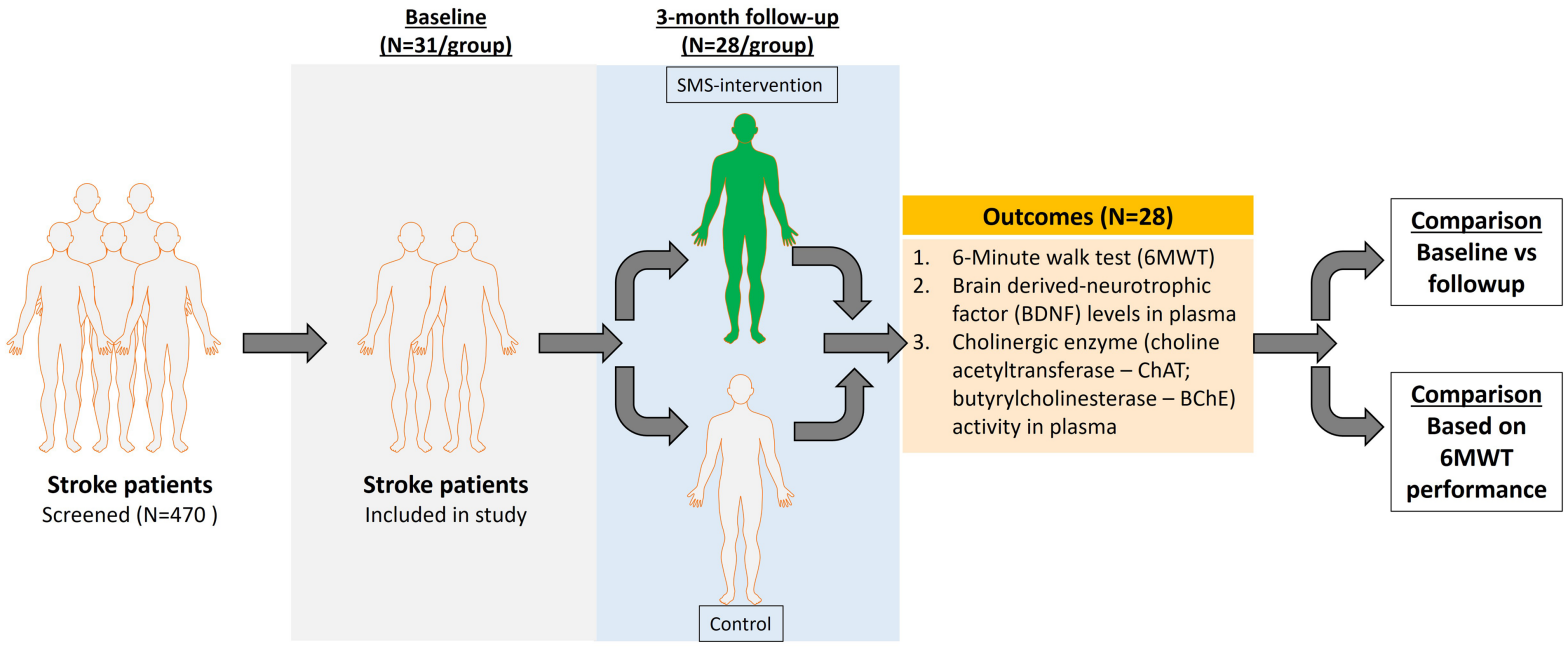
Schematic representation of the study.

This study, summarized in Figure 6, shows the relevance and feasibility of using BDNF and cholinergic index as biomarkers for post-stroke recovery. Exercise could resist the decline in BDNF levels post-stroke. Whether cholinergic signaling plays a role in enhanced walking performance or increased walking elevates cholinergic function in post-stroke patients needs further validation.

## 6 Limitations and strengths

This study presents an exploratory analysis aimed at gaining insight into the potential interrelationships between stroke, brain-derived neurotrophic factor (BDNF), and the modulation of key components of the cholinergic system. We conducted a *post hoc* analysis on a small cohort of patients.

One of the main limitations of our study is the absence of brain MRI data, which restricts our ability to determine whether improvements in the 6-min walk test (6MWT) and changes in the observed biomarkers are associated with structural brain recovery following the 3-month intervention period. Additionally, the relatively short duration of follow-up further limits the interpretation of long-term outcomes.

We could not establish whether patients not receiving the SMS instructions were devoid of exercise regimens other or similar than the ones recommended through the SMS instructions. Moreover, stratification based on 6MWT considers only the improvement of ≥34 m versus <34 m irrespective of whether they received SMS reminders or not, thereby only considering the clinical outcome with the parameters measured in this study.

*Generalizability*: This model of text messaging may not apply to individuals with more limited mobility after stroke, those with aphasia, or those with cognitive impairments who return home.

*Potential bias*: Random missing data, inability to fully maintain the assessor blinded at follow-up, challenges with using the training diary, and limited use of the pedometers.

Missing data can reduce statistical precision, increase standard error, and introduce bias if the missing data is not random. This makes it more difficult to draw valid conclusions and generalize the results to a broader population. Missing data in this study was random.

A key strength of this study is its randomized controlled design, combined with the use of real-world data. The intervention itself is also a notable advantage—it can be integrated alongside existing rehabilitation services, require no personnel, is easily accessible regardless of location, and is cost-effective. Additionally, the study included both men and women across a broad age range, enhancing the generalizability of the findings. Another strength is the lower-than-expected dropout rate, which supports the feasibility and acceptability of the intervention.

## Data Availability

The raw data supporting the conclusions of this article will be made available by the authors, without undue reservation.

## Footnotes

^1^ www.intime.nu
