# Supplementary material for: Plasma cholinergic markers are associated with post-stroke walking recovery—revisiting the STROKEWALK study
Source: Front Neurol. 2025 May 30;16:1568401. doi: 10.3389/fneur.2025.1568401 (PMC12162941; doi:10.3389/fneur.2025.1568401)
Supplement: Supplementary file 2 [file Supplementary_file_1.docx]

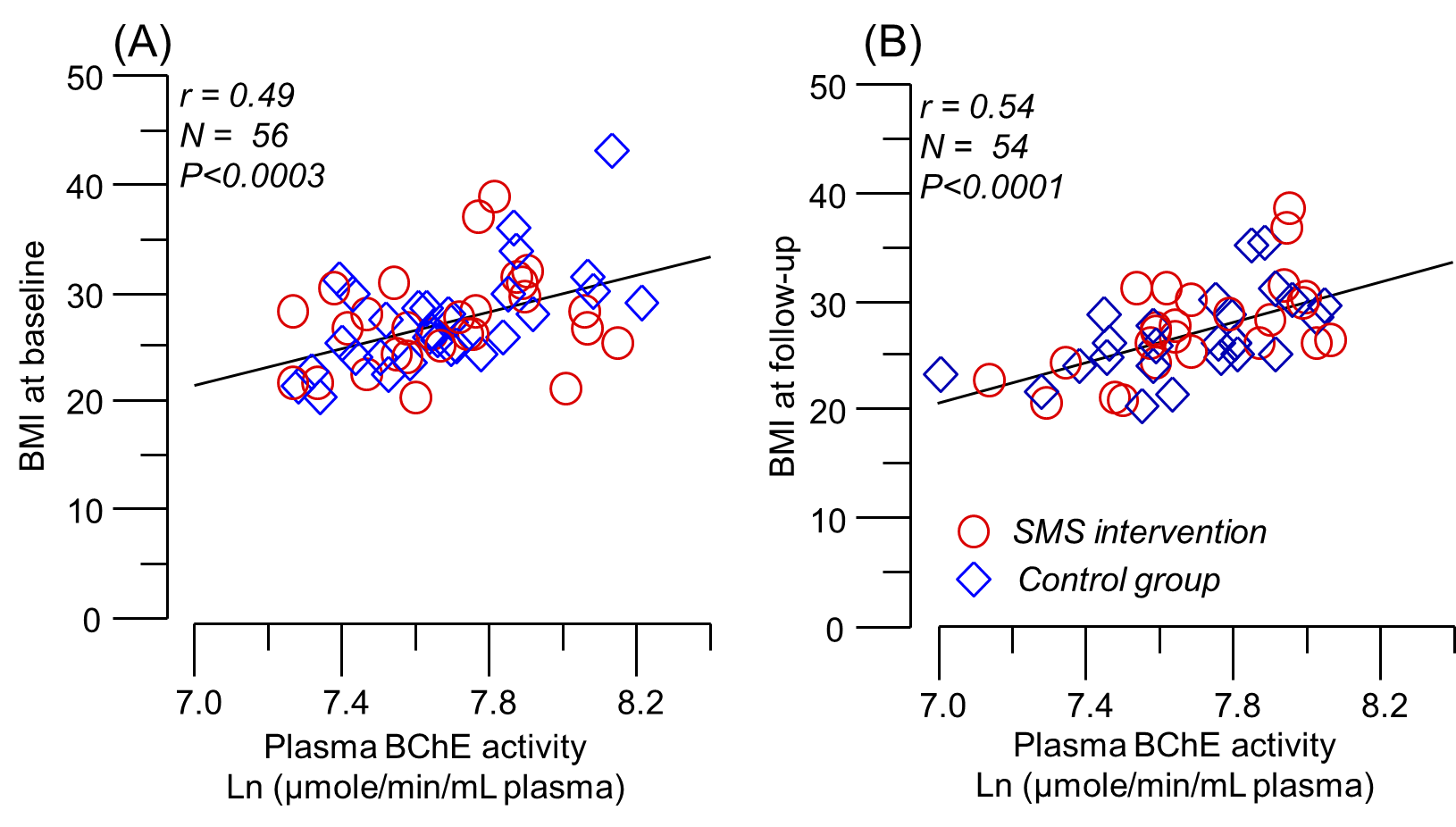


**Supplementary Figure 1.** Butyrylcholinesterase (BChE) activity correlated with body-mass index (BMI). A) shows the correlation between BMI and BChE activity (Ln) among all the patients at baseline. B) shows the correlation between BMI and BChE activity (Ln) among all the patients at 3-month follow-up.
